# Effects of Liver Resection on Hepatic Short-Chain Fatty Acid Metabolism in Humans

**DOI:** 10.1371/journal.pone.0166161

**Published:** 2016-11-11

**Authors:** Evelien P. J. G. Neis, Johanne G. Bloemen, Sander S. Rensen, Joost R. van der Vorst, Maartje A. van den Broek, Koen Venema, Wim A. Buurman, Cornelis H. C. Dejong

**Affiliations:** 1 TI Food and Nutrition, 6709 PA Wageningen, The Netherlands; 2 Department of Surgery, NUTRIM School for Nutrition and Translational Research in Metabolism, Maastricht University Medical Centre, 6229 HX Maastricht, The Netherlands; 3 Department of Surgery, Leiden University Medical Centre, 2333 ZA Leiden, The Netherlands; 4 Beneficial Microbes Consultancy, Wageningen University, 6708 PB Wageningen, The Netherlands; Bambino Gesù Children's Hospital, ITALY

## Abstract

**Aim:**

To determine whether acute loss of liver tissue affects hepatic short-chain fatty acid (SCFA) clearance.

**Methods:**

Blood was sampled from the radial artery, portal vein, and hepatic vein before and after hepatic resection in 30 patients undergoing partial liver resection. Plasma SCFA levels were measured by liquid chromatography-mass spectrometry. SCFA exchange across gut and liver was calculated from arteriovenous differences and plasma flow. Liver volume was estimated by CT liver volumetry.

**Results:**

The gut produced significant amounts of acetate, propionate, and butyrate (39.4±13.5, 6.2±1.3, and 9.5±2.6 μmol·kgbw^-1^·h^-1^), which did not change after partial hepatectomy (p = 0.67, p = 0.59 and p = 0.24). Hepatic propionate uptake did not differ significantly before and after resection (-6.4**±**1.4 vs. -8.4**±**1.5 μmol·kgbw^-1^·h^-1^, p = 0.49). Hepatic acetate and butyrate uptake increased significantly upon partial liver resection (acetate: -35.1**±**13.0 vs. -39.6**±**9.4 μmol·kgbw^-1^·h^-1^, p = 0.0011; butyrate: -9.9**±**2.7 vs. -11.5**±**2.4 μmol·kgbw^-1^·h^-1^, p = 0.0006). Arterial SCFA concentrations were not different before and after partial liver resection (acetate: 176.9±17.3 vs. 142.3±12.5 μmol/L, p = 0.18; propionate: 7.2±1.4 vs. 5.6±0.6 μmol/L, p = 0.38; butyrate: 4.3±0.7 vs. 3.6±0.6 μmol/L, p = 0.73).

**Conclusion:**

The liver maintains its capacity to clear acetate, propionate, and butyrate from the portal blood upon acute loss of liver tissue.

## Introduction

Short-chain fatty acids (SCFA, i.e. acetate, propionate, and butyrate) have lately attracted considerable attention since they are thought to underlie the effect of gut bacteria on body weight and metabolism.[1] These organic acids constitute the main products of bacterial fermentation of indigestible carbohydrates in the human colon.[2] Once produced, SCFA are for approximately 90% metabolized inside colonocytes. Another 5% is excreted with feces, and the remainder is thought to be released into the portal vein.[3, 4] In the liver, acetate and butyrate are metabolized to acetyl-coA before entering the tricarboxylic acid (TCA) cycle to generate ATP and NADH.[5] Propionate, on the other hand, functions primarily as a precursor of gluconeogenesis in liver cells.[6]

Currently, there is growing interest in functional foods that affect the composition of gut microbiota, and which may lead to the generation of these SCFA.[7, 8] Indeed, SCFA generated by the intestinal fermentation of dietary fibers seem to have many positive actions on health in terms of (body weight regulation, gut micromorphology, and insulin homeostasis.[3, 9–12] As a result of the increasing evidence for a potential role of SCFA as a metabolic tool, various studies have reported on beneficial effects of SCFA or SCFA precursor supplementation in (pre-) clinical settings.[13–15] We recently reported on a possible clinical application of butyrate to increase intestinal anastomotic strength.[16] Whereas these studies support the use of SCFA for improving gut health in man, it is pivotal to better understand human SCFA metabolism before therapeutic SCFA supplementation can be widely implemented, particularly given that high systemic concentrations of especially propionate and butyrate are toxic.[17, 18]

Our group has previously shown that release of intestinal SCFA appears to be equaled by hepatic uptake [19], even in patients with a cirrhotic dysfunctional liver.[19] However, a potential drawback of the latter study was the possible shunting of blood from the portal to the systemic circulation in patients with liver cirrhosis, making exact quantification of hepatic SCFA metabolism in this population difficult.

To address this problem, we now studied SCFA metabolism in a controlled situation of acute loss of liver function where shunting does not play a role, i.e. surgical removal of major parts of the liver.

## Materials and Methods

### Study population

We included thirty patients planned to undergo liver resection to remove colorectal cancer metastasis at Maastricht University Medical Centre^+^ (MUMC^+^). All patients provided informed consent. Patients with known parenchymal liver disease, inborn errors of metabolism, diabetes mellitus type I, and/or use of antibiotics four weeks prior to the operation were excluded from the study. All patients were on a stable, Western diet. Immediately preoperatively, patients received a single intravenous dose of 2200 mg amoxicillin/clavulanic acid as antibiotic prophylaxis.

The study was approved by the Local Medical Ethics Committee of Maastricht University Medical Center and was performed in accordance with the ethical standards of the Helsinki Declaration of 1975. Written informed consent was obtained from all subjects before participation in this study.

### Study protocol

Anaesthesia was performed according to institutional routines as has been described previously.[20, 21] Briefly, the procedure included placement of two peripheral venous catheters, an epidural catheter for per- and postoperative analgesia, an arterial line, and a central venous line. Anesthesia was maintained using sevoflurane and propofol. Liver resections were performed as described before and classified as major (i.e. ≥ 3 segments) or minor (< 3 segments).[20] Liver resection started with mobilization of the liver, whereupon intraoperative ultrasound (Aloka, Zug, Switzerland) determined the definitive surgical procedure. Liver transection was performed using a Cavitron Ultrasonic Surgical Aspirator (CUSA, system 200 Macrodissector; Cavitron Surgical Systems, Stamford, CT). Argon beam coagulation (Force GSU Systems; Valleylab, Boulder, CO), clips and sutures were used to achieve hemostasis. When the portal and hepatic veins were exposed (mostly within one hour after skin incision, but before liver transection), blood was drawn from the portal vein and a hepatic vein by direct puncture simultaneously with arterial blood sampling, as described before.[20] Directly after hepatic transection, blood was sampled again from the portal vein, a hepatic vein and the radial artery simultaneously. Blood was collected in EDTA vacuum tubes (BD Vacutainer, Franklin Lakes, NJ) and placed on ice. Blood was centrifuged at 3,500 g and plasma was stored at -80^0^ C until analysis. Finally, the liver resection specimens were weighed.

### Sample preparation and liquid chromatography-mass spectrometry (LC-MS)

Deproteinization and subsequent preparation of plasma samples for analysis of SCFA was performed as recently reported.[22] Briefly, plasma samples were deproteinized using methanol, after which they were centrifuged at 50,000 g for 10 minutes at 4°C. The clear supernatant was transferred to a 300 μl glass micro-insert into a WISP-style vial. Analysis was performed using LC-MS. SCFA concentrations were determined using the external standard method by calibration curves of SCFA. The detection limits for acetate, propionate, and butyrate were 0.1, 0.05, and 0.05μmol/L, respectively. The coefficients of variance were 4.2%, 9.8%, and 5.1% for acetate, propionate, and butyrate, respectively.

### CT-volumetry

Liver volumetry was performed in a subset of 16 patients using open source software OsiriX^®^, as described before.[23] Briefly, series of axial images in the portal venous phase from the preoperative computed tomography (CT) scans were used for volumetry. A slice thickness of approximately 3–5 mm, depending on the CT-scanner was used. The outline of the total liver, future resection specimen, and tumors were traced manually on each slice with specific tools belonging to OsiriX^®^. Whereas intrahepatic vascular and biliary structures were included, the gall bladder and the inferior caval vein were excluded for all slices. After selecting all regions of interest within one series, total liver volume, resection volume, and metastases volumes were calculated. Then, 3D images were created and virtual resections were performed according to the treatment plan. Functional volume was calculated as total volume minus tumor volume. Subsequently, these volumes were used to estimate the remnant liver volume in order to assess differences in SCFA clearance per gram functional liver tissue pre- and post- hepatic resection.

### Flux calculations

Arteriovenous differences (ΔAV) and net organ fluxes (flow * ΔAV) were calculated across the liver, portal drained viscera (PDV), and splanchnic area. Plasma flows for flux calculations were derived from previously performed measurements in a similar patient group.[20] As we demonstrated before that total hepatic plasma flow does not change after major hepatectomy [24], the same plasma flow data were used before and after resection. The corresponding AV differences were calculated as follows: ΔAV_PDV_ = [PV]–[A] and ΔAV_splanchnic area_ = [HV]–[A]. Fluxes were calculated as F_PDV_ = portal plasma flow * [PV-A], F_splanchnic_ = splanchnic plasma flow * [HV]-[A], F_liver_ = F_splanchnic_-F_PDV_. In these equations [PV], [A], and [HV] indicate portal venous, radial artery, and hepatic venous concentrations respectively, whereas F_splanchnic,_ F_liver,_ F_PDV_ denote splanchnic flux, liver flux, and PDV flux, respectively. Positive fluxes indicate net release and negative fluxes indicate net uptake. To estimate hepatic functional reserve, hepatic SCFA exchange was also calculated per gram of liver tissue using the volumetry data and assuming 1 mL corresponds with 1 gram of liver tissue.

### Statistical analysis

Data are presented as mean (SEM). To test if fluxes were statistically different from zero, the nonparametric Wilcoxon signed-rank test was used with a hypothetical value of zero. The nonparametric Wilcoxon signed-rank matched pairs test was used to test if there were differences in fluxes and concentrations before and after partial hepatectomy. Correlations were calculated with Spearman’s correlation coefficients. A p-value of <0.05 was considered statistically significant. For statistical analysis, Prism 5.0 for Windows (Graphpad Software Inc. San Diego, CA) was used.

## Results

### Patient population

Baseline characteristics of the thirty patients included in the study are presented in Table 1. Sampling was performed in all patients according to protocol. Thirteen patients underwent major liver resection, whereas 17 patients underwent minor liver resection. The median time required for transection was 110 minutes.

**Table 1:** Baseline characteristics (n = 30).

Sex (Male)		20
Age (years)		62 (41–79)
BMI (kg/m^2^)		25.1 (20.3–33.3)
Neoadjuvant chemotherapy		20
Indication	Colorectal liver metastases	26
	Cholangiocarcinoma	1
	Carcinoid	1
	HCC	2
Type of liver resection	Minor	17
	Major	13
Colonic surgery in the past	No colonic surgery	8
	Right sided	8
	Left sided	13
	Transverse colon	1
Plasma flows (mL/min)	Portal vein	320 (42)
	Hepatic artery	110 (23)
	Splanchnic	430 (47)

BMI: body mass index, HCC: hepatocellular carcinoma. Age and BMI are expressed as median (range). Plasma flows are expressed as mean (SEM)

### SCFA production by the gut is not affected by partial liver resection

**Fig 1:**
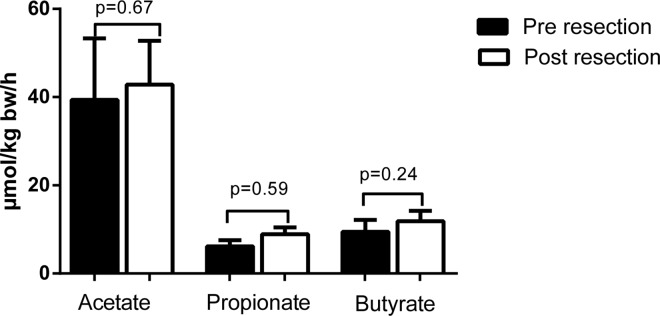
Short chain fatty acid fluxes across the gut.

The gut produced significant amounts of acetate, propionate, and butyrate before liver transection, as evidenced by portal venous concentrations exceeding the arterial concentrations. The corresponding fluxes were 39.4±13.5, 6.2±1.3, and 9.5±2.6 μmol·kg bw^-1^·h^-1^ for acetate, propionate, and butyrate, respectively (p<0.01 for all SCFA, Fig 1). As expected, the production of acetate, propionate, and butyrate by the gut directly after partial liver resection was not statistically different from baseline production, with corresponding fluxes of 42.8±9.6, 8.9±1.5, and 11.9±2.3 μmol·kg bw^-1^·h^-1^, respectively (p = 0.67, p = 0.59, and p = 0.24 respectively; Fig 1).

### Increased hepatic acetate and butyrate uptake after partial liver resection

**Fig 2:**
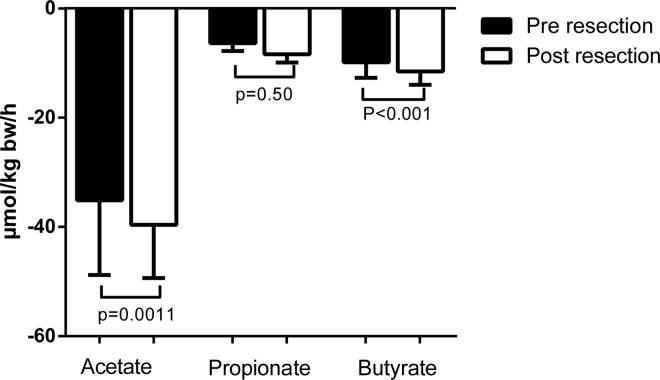
Short chain fatty acid fluxes across the liver.

Acetate, propionate, and butyrate were all taken up by the liver both before (acetate: -35.1**±**13.0 μmol·kg bw^-1^·h^-1^, p<0.01, propionate: -6.4**±**1.4 μmol·kg bw^-1^·h^-1^, p<0.001, butyrate: -9.9±2.7 μmol·kg bw^-1^·h^-1^, p<0.001) and after partial liver resection (acetate: -39.6±9.4 μmol·kg bw^-1^·h^-1^, p<0.001, propionate: -8.4±1.5 μmol·kg bw^-1^·h^-1^, p<0.001, butyrate: -11.5**±**2.4 μmol·kg bw^-1^·h^-1^, p<0.0001; Fig 2). Whereas the increases in acetate and butyrate uptake after liver resection were significant (p = 0.0011, p<0.0001, respectively), the increase in propionate uptake was not (p = 0.50).

### Splanchnic SCFA fluxes and systemic concentrations

**Fig 3:**
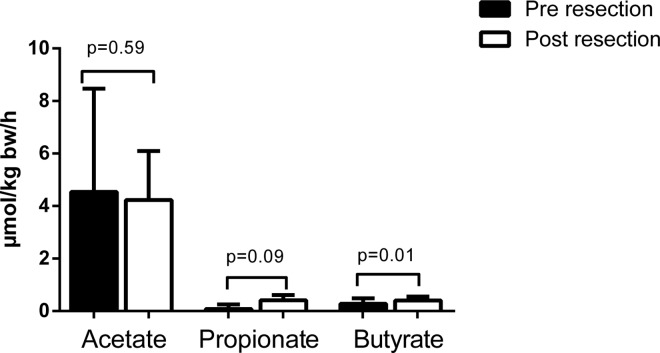
Short chain fatty acid fluxes across the splanchnic area.

Since acetate and propionate release from the gut equalled hepatic uptake both before and after partial liver resection, there was no significant acetate or propionate release from the splanchnic area at either time point. The corresponding splanchnic fluxes were 4.5±3.8 μmol·kg bw^-1^·h^-1^ before and 4.2±1.8 μmol·kg bw^-1^·h^-1^ after partial liver resection for acetate, and 0.1±0.2 μmol·kg bw^-1^·h^-1^ before and 0.4±0.2 μmol·kg bw^-1^·h^-1^ after partial liver resection for propionate (both p>0.05, Fig 3). A small but significant release of butyrate from the splanchnic area was found only after partial liver resection (0.4±0.1 μmol·kg bw^-1^·h^-1^, p = 0.0103). Arterial acetate, propionate, and butyrate concentrations were not significantly different before versus immediately after partial liver resection; Table 2.

**Table 2:** Arterial SCFA concentrations before and after resection (n = 30).

	Before	After	p-value
**Acetate**	176.9 (17.3)	142.3 (12.5)	0.18
**Propionate**	7.2 (1.4)	5.6 (0.6)	0.38
**Butyrate**	4.3 (0.7)	3.6 (0.6)	0.73

Concentrations in μmol/L

### No effect of partial liver resection on SCFA uptake per gram liver tissue

Before resection, mean total liver volume was 1692±88 mL and mean functional liver volume (total liver volume minus tumor volume) was 1584±116 mL. Mean estimated remnant liver volume after resection was 1186±106 mL. The resection specimens weighed on average 475±60 g, which was equivalent to 28% of total liver volume. Uptake of acetate, propionate, and butyrate by the liver before partial resection did not correlate with the percentage functional remnant liver volume (r_s_ = 0.32 for acetate (p = 0.20), r_s_ = 0.22 for propionate (p = 0.38) and r_s_ = 0.21 for butyrate (p = 0.39). Similarly, after partial resection, the uptake of acetate, propionate, and butyrate by the liver was not correlated with the percentage functional remnant liver volume (r_s_ = -0.08 for acetate (p = 0.73), r_s_ = 0.16 for propionate (p = 0.51) and r_s_ = 0.13 for butyrate (p = 0.60). Furthermore, no differences were found between arterial acetate, propionate, and butyrate concentrations in patients that underwent a minor resection compared to patients that underwent a major resection; Table 3. When assessing the flux per gram liver tissue before and after liver resection, no significant change in uptake per gram liver tissue was seen for any SCFA (p = 0.87 for acetate, p = 0.88 for butyrate and p = 0.12 for propionate).

**Table 3:** Arterial concentrations before and after minor (n = 17) and major resection (n = 13).

	Minor resection			Major resection		
	Before	After	p-value	Before	After	p-value
**Acetate**	189.9 (27.8)	155.4 (16.9)	0.97	158.5 (17.61)	124.8 (19.8)	0.27
**Propionate**	7.7 (2.2)	5.9 (0.9)	0.90	6.7 (1.5)	5.2 (1.0)	0.70
**Butyrate**	5.2 (1.1)	4.0 (0.8)	0.27	3.1 (0.6)	3.0 (0.6)	0.20

Concentrations in μmol/L

## Discussion

The present study was undertaken to investigate the effect of partial liver resection, as a model for controlled loss of liver function, on interorgan exchange of short chain fatty acids (SCFA). Our data confirm that the gut produces significant amounts of acetate, propionate, and butyrate. Intestinal production of acetate exceeds the production of butyrate which, in turn, is higher than the production of propionate. These gut derived SCFA are subsequently to a large extent taken up by the liver. Hepatic uptake of acetate and butyrate even increases after partial liver resection.

Together with data from our previous studies [19], the present data indicate that the liver is able to take up acetate, propionate and butyrate proportionally to gut production, both before and after partial liver resection. These findings are in concordance with previous work by our group showing excess hepatic capacity in e.g. hepatic urea synthesis and ammonia clearance.[20]

Complete hepatic clearance of the main SCFA acetate, propionate, and butyrate (even after partial hepatectomy) is important to avoid the possible toxicity of SCFA. However, it may also be considered as less beneficial given recent data on the metabolic signaling activities of these SCFA. In fact, an enteroendocrine pathway has been proposed by which SCFA control gut hormone expression. SCFA are ligands for G-protein-coupled receptors (GPCRs) GPR43 and GPR41 expressed on enteroendocrine L-cells.[25] In response to SCFA activation of these GPCRs, the gut hormones glucagon-like peptide (GLP-1) and Peptide YY (PYY) are secreted.[26] PYY subsequently regulates intestinal motility and thereby nutrient absorption from the gut, whilst GLP-1 affects satiety.[27] The secretion of GLP-1 is reduced in type 2 diabetes and seems to be reduced in obese subjects as well.[28] Furthermore, mice lacking GPR43 exhibit reduced SCFA-triggered GLP-1 secretion and an impairment of glucose tolerance.[29] More recently, Kimura et al. demonstrated that GPR43 links SCFA production by gut microbiota to host energy homeostasis.[30] They showed that GPR43- deficient mice are obese on a normal diet, whereas mice overexpressing GPR43 specifically in adipose tissue remain lean even when fed a high-fat diet. Besides, SCFA have been shown to influence obesity-induced chronic low-grade inflammation. In addition, acetate, propionate, and butyrate can directly decrease the secretion of adipose tissue-derived proinflammatory cytokines and chemokines. Butyrate, in particular, can also indirectly influence proinflammatory cytokine and chemokine production by influencing signaling pathways like the nuclear factor-kB pathway and by inhibition of histone deacetylases.[31, 32] Butyrate is part of a well-known class of epigenetic regulators known as histone deacetylase inhibitors (HDACi), which modulate the accessibility of genes to transcription factors. Hence, dietary manipulation of histone structure and function of critical genes associated with physiologic and pathologic processes [33] may be a solution to the puzzle of the relation between dietary fiber and the prevention and treatment of different diseases. In this regard, there is a growing interest in butyrate as its impact on epigenetic mechanisms will lead to potential clinical implications.[34]

SCFA might also enhance the intestinal barrier function, further supporting their anti-inflammatory potential. In several studies using intestinal cell lines, SCFA (particularly butyrate), have been shown to improve epithelial barrier function and gut permeability by modulating expression of tight junction proteins and mucins. [35, 36] As such, SCFA, and the microbiota that produce them, are considered to modulate human metabolism by acting as signaling molecules [37–39].

Whereas hepatic acetate and butyrate uptake was increased after liver resection, no significant changes appeared to occur in the uptake per gram liver tissue. This might be related to the fact that the uptake per gram liver tissue could only be calculated in a subgroup of patients and the substantial variation in hepatic SCFA uptake between patients. Our data further showed that the uptake of acetate, propionate, and butyrate by the liver was not correlated with the percentage functional remnant liver volume. This may indicate that the magnitude of hepatic SCFA clearance is dependent on gut production, i.e. SCFA availability. Of note, it should be taken into account that the patients were fasting overnight before the operation, so the intestinal SCFA production was lower during the sampling than the usual average production rate. In a sheep model, Bergman et al.[40] found that acetate concentrations were indeed significantly lower in the fasted state compared to the fed state, despite the intensive microbial SCFA production in the rumen of ruminants. In contrast to this study in which the sheep were fasted for three days, the patients in our study had just an overnight fast which may suggest that the influence of fasting on SCFA availability in our study was less pronounced.

Our current data obtained in a unique model of acute loss of liver function underscore the large capacity of the liver to metabolize SCFA released from the gut. It should, however, be taken into consideration that it only reflects a short term acute loss of liver function as blood samples were taken immediately after the partial liver resection. Additional intervention studies must be performed to ensure that supplementation of SCFA in patients is safe. Of note, administering precursors of SCFA (i.e. inulin or fructo-oligosaccharides) has been reported to lead to increased intraluminal SCFA concentrations, but systemic changes in SCFA concentrations have not been reported to date.[41, 42]

## Conclusion

This in vivo study in humans confirmed that the gut produces significant quantities of the main SCFA acetate, propionate, and butyrate. Acute hepatic tissue loss did not influence systemic concentrations of SCFA, implying that the liver has a large reserve capacity to metabolize propionate, acetate, and butyrate to prevent any increase of arterial concentrations. This was underscored by the increased hepatic uptake of acetate and butyrate after partial liver resection which, in turn, may be interpreted as circumstantial evidence for the safety of SCFA supplementation even in patients with limited liver tissue.
